# Novel read‐through fusion transcript Bcl2l2‐Pabpn1 in glioblastoma cells

**DOI:** 10.1111/jcmm.17481

**Published:** 2022-07-27

**Authors:** Lin Zhang, Dan Wang, Xiao Han, Xiaoxiao Guo, Yuanyuan Cao, Ying Xia, Dianshuai Gao

**Affiliations:** ^1^ Department of Neurobiology and Anatomy, Xuzhou Key Laboratory of Neurobiology Xuzhou Medical University Xuzhou China; ^2^ School of Nursing Xuzhou Medical University Xuzhou China; ^3^ School of Medical Information and Engineering Xuzhou Medical University Xuzhou China; ^4^ Nanjing Medical University Nanjing China

**Keywords:** Bcl2l2‐Pabpn1, fusion transcript, glioblastoma, read‐through, RNA‐seq

## Abstract

Read‐through fusion transcripts have recently been identified as chimeric RNAs and have since been linked to tumour growth in some cases. Many fusion genes generated by chromosomal rearrangements have been described in glioblastoma. However, read‐through fusion transcripts between neighbouring genes in glioblastoma remain unexplored. We performed paired‐end RNA‐seq of rat C6 glioma cells and normal cells and discovered a read‐through fusion transcript Bcl2l2‐Pabpn1 in which exon 3 of Bcl‐2‐like protein 2 (Bcl2l2) fused to exon 2 of Polyadenylate‐binding protein 1 (Pabpn1). This fusion transcript was found in both human glioblastoma and normal cells. Unlike other fusions reported in glioblastoma, Bcl2l2‐Pabpn1 appeared to result from RNA processing rather than genomic rearrangement. Bcl2l2‐Pabpn1 fusion transcript encoded a fusion protein with BH4, BCL and RRM domains. Functionally, Bcl2l2‐Pabpn1 knockdown by targeting its fusion junction decreased its expression, and suppressed cell proliferation, migration and invasion in vitro. Mechanistically, Bcl2l2‐Pabpn1 blocked Bax activity and activated PI3K/AKT pathway to promote glioblastoma progression. Together, our work characterized a glioblastoma‐associated Bcl2l2‐Pabpn1 fusion transcript shared by humans and rats.

## 1 INTRODUCTION

Glioblastoma is the most malignant brain tumour with strong proliferative and infiltrative abilities that cannot be cured by traditional therapy due to the great differences in cellular and molecular levels.^1^, ^2^, ^3^ WHO included molecular characteristics to be used for glioma classification for the first time in 2016.^4^ As a result, demonstrating the molecular characteristics of glioblastoma would benefit its treatment and diagnosis. As important biomarkers of oncogenesis, fusion genes have been identified as valuable diagnostic and prognostic indicators, as well as therapeutic targets.^5^, ^6^, ^7^ With current sequencing technologies and bioinformatics approaches, in addition to haematological diseases, a list of fusion genes have been identified in various solid tumours, such as lung cancer,^7^, ^8^ prostate cancer^9^, ^10^ and breast cancer.^11^, ^12^ Also, numerous fusion genes have been discovered in glioblastoma: FIG‐ROS,^13^ PTPRZ1‐MET,^14^ HMGA2‐EGFR^15^ and FGFR‐TACC.^16^ Currently, transcriptome and genome analysis are enabling the systematic identification of known or novel fusions in solid tumours.^17^

In addition to fusion genes generated by chromosomal rearrangements at the DNA level,^18^, ^19^ such as translocation,^20^ insertion, inversion^21^ and deletion,^22^ fusion transcripts can be caused by transcription‐induced chimeric pre‐mRNA through trans‐^23^ or cis‐splicing^24^ at the RNA level. These fusions can be produced between two genes which are 50 kb apart but more often are very close.^25^ Read‐through fusion transcripts, a particular class of chimeric transcripts, can occur in the absence of genomic aberrations.^18^, ^26^ Generally, this type of chimeric RNAs arise between neighbouring genes in the same orientation through last and first exon skipping, where the upstream and downstream genes lack the last and first exon, respectively.^27^ Some of the read‐through fusions have been discovered in prostate cancer,^28^ gastric cancer^27^ and breast cancer,^26^ and are related to tumour progression. However, read‐through fusions have not been identified in glioblastoma.

Here, we discovered a read‐through fusion transcript Bcl2l2‐Pabpn1 using paired‐end RNA‐seq. Bcl2l2 (Bcl‐2‐like protein 2) is also known as Bcl‐w (B cell lymphoma‐w), and a pro‐survival member of the Bcl‐2 family.^29^ Bcl2l2, an oncogene, has been reported to promote cell proliferation, migration and invasion in various tumours,^30^, ^31^, ^32^ including glioblastoma.^33^, ^34^ Although this fusion was reported in human cells in the database (provided by RefSeq, Dec 2010), it has not been characterized in rat cells and there is no investigation about the roles of Bcl2l2‐Pabpn1 in cancers. In this study, we identified Bcl2l2‐Pabpn1 fusion transcript not only in glioblastoma cells but also in normal cells, and this fusion is shared by humans and rats. This chimeric RNA is translated into a fused protein and exhibited tumour‐promoting capacity.

## 2 MATERIALS AND METHODS

### 2.1 Animals

Newborn Sprague–Dawley (SD) rats (days 2–3) for rat's astrocytes acquisition were obtained from the Experimental Animal Center of Xuzhou Medical University (China), and their gender was random. All experiments involving animals were carried out according to the Guide for the Care and Use of Laboratory Animals (NIH Publication Nos. 80–23, revised 1996), ensuring that their suffering is minimized. This work was approved by the Animal Ethics Committee of Xuzhou Medical University.

### 2.2 Cell lines and cell culture

Glioblastoma cell lines (human: U87, U251, A172, LN229, U118 and T98G; rat: C6) and normal human astrocytes (HA) were purchased from the Cell Bank of Chinese Academy of Sciences and Science Research Laboratories, respectively. These cells were authenticated by STR profiling. Rat primary astrocytes (AST) were prepared in our laboratory as described previously.^35^ Human glioblastoma cells were cultured in Dulbecco's Modified Eagles medium (DMEM; Gibco) with 10% fetal bovine serum (FBS; Gibco), and HA cells were cultured in Astrocyte Medium (Science Research Laboratories) with 10% FBS and 1% SMCGS. Rat C6 and AST cells were cultured in DMEM/F12 (Gibco) containing 10% FBS.

### 2.3 RNA‐seq

Total RNA from rat C6 and AST cells was extracted with TRIzol reagent (Invitrogen, Life Technologies). RNA integrity was validated by Agilent 2100 (Agilent Technologies), and quantification and quality of RNA were determined by Qubit 2.0 (Life Technologies) and Nanodrop (IMPLEN), respectively. Purified RNA was used to construct the RNA‐seq libraries using NEBNext®ultra™ RNA Library Preparation Kit (NEB) according to the manufacturer's instructions. The libraries were validated with the Agilent 2100 to inspect the insert size, and then sequenced on the Illumina platform using 150 bp paired‐end RNA‐seq (Novogene). High‐quality reads obtained were aligned to the reference genome using TopHat2 software (v2.0.12). Then, FusionMap was used to identify potential fusion transcripts based on the resulting alignments.^36^ The parameters used were as follows: unique cutting position count >2, seed count ≥3, canonical splice pattern and empty filter.

### 2.4 PCR analysis and Sanger sequencing

RNA isolated from glioblastoma and normal cells was reverse‐transcribed into cDNA with PrimerScript™ RT reagent Kit (Takara). PCR primers flanking the fusion junction were designed to amplify the Bcl2l2‐Pabpn1 read‐through fusion transcript. For PCR analysis to evaluate the presence of Bcl2l2‐Pabpn1, the following primers were used: human‐(F)5′‐TGGTGGCAGACTTTGTAGG‐3′, (R)5′‐CCGGTCTGTTGTGCTGATG‐3′; rat‐(F)5′‐TAGTGGCTGACTTTGTAGG‐3′, (R)5′‐TGTCACAGAGTATAGTCACC‐3′. The PCR products were analysed by gel electrophoresis. After the PCR products were purified, Sanger sequencing was conducted to validate their sequences. For qPCR to determine the efficiency of Bcl2l2‐Pabpn1 knockdown, the primers are as follows. Primer1: (F)5′‐AGAGTGTCAACAAGGAGATGGA‐3′, (R)5′‐TCTGCTTCTCTACCTCGTT CTG‐3′; Primer2: (F)5′‐GCCGCCTTGTAGCCTTCTT‐3′, (R)5′‐CTTCTTCCTCCA TCTCCCTGAC‐3′. The relative expression was calculated using the $2^{-\Delta \Delta C_{T}}$ method.

### 2.5 Western blotting analysis

Glioblastoma and normal cells were lysed by RIPA buffer containing PMSF (1:100; Beyotime Biotechnology) for 30 min. The lysed cells were centrifuged at 12,000 *g* for 30 min at 4°C, and then, the supernatant was collected. The protein samples were loaded into 10% SDS‐PAGE gel and transferred to PVDF membranes. The membranes were blocked by 5% skimmed milk for 2 h at room temperature and incubated with primary antibodies at 4°C overnight. After washing by TBST three times (5 min each time), blots were incubated with corresponding secondary antibodies for 2 h in the dark at room temperature. Finally, the membranes were cleaned with TBST three times, and then imaged. The antibodies used are shown in Table S1.

### 2.6 Construction and transfection

Stable cell lines for Bcl2l2‐Pabpn1 knockdown were constructed by Obio Technology. According to the nucleotide sequences of Bcl2l2‐Pabpn1 read‐through fusion transcript, two shRNAs were designed to target the fusion junction and a non‐targeting shRNA was used as a control. We successfully designed shRNA#1 (GGGAGCTGGAAGCTATCAA) using BLOCK‐iT™ RNAi Designer of ThermoFisher. The software did not provide other valid shRNAs, so, we designed additional shRNA#2 (GGCTGGGAGCTGGAAGCTA) targeting fusion junction of Bcl2l2‐Pabpn1 manually, and the non‐targeting shRNA (TTCTCCGAACGTGTCAC GT). U87 and U251 cells were transfected with lentivirus for 72 h, where 1 μg/ml puromycin was added and maintained. After 2 weeks, cells were screened to form stable Bcl2l2‐Pabpn1 knockdown cell lines, which would be used in the following functional experiments.

### 2.7 Cell Counting Kit‐8 assay

Cell Counting Kit‐8 assay (CCK8) was conducted to examine the cell viability. Stable U87 and U251 cells for Bcl2l2‐Pabpn1 knockdown were seeded into 96‐well plates (1.5 × 10^3^ cells/well) and cultured in DMEM with 10% FBS overnight. Then, 10 μl CCK8 dye (Dojindo Laboratories) was added at the indicated time‐points (0, 24, 48, 72, 96 h) and incubated for 2 h at 37°C. Then, the OD values at 450 nm were examined using a microplate reader (Spark 10M; TECAN). The first CCK8 result was regarded as starting value (0 h). All experiments are performed in triplicates.

### 2.8 Colony formation assay

Three hundred cells were seeded in 24‐well plates and cultured for 12 days. After being washed by PBS three times, cells were fixed with methanol, stained with 0.1% crystal violet and then photographed. All experiments are performed in triplicates.

### 2.9 EdU assay

Cell proliferation was examined by EdU assay using EdU detection kit (Beyotime) according to the manufacturer's protocols. Briefly, after incubation for 2 h in DMEM containing 20 μM EdU, cells were fixed using 4% paraformaldehyde, permeabilized using 1% Triton X‐100 and stained with Apollo and Hochest in dark. Then, cells were imaged by fluorescence microscopy and counted for EdU‐positive cells by Image J. All experiments are performed in triplicates.

### 2.10 Cell cycle assay

Cell cycle distribution was analysed by flow cytometry. After U87 and U251 cells were collected and washed with precooled PBS twice, they were fixed in 70% ethanol at 4°C for 1 h. Subsequently, the cells were centrifuged at 1000 ***g*** for 5 min and washed with precooled PBS twice. Following incubation in 20 μg/ml RNase A in the dark at 37°C for 30 min to digest RNA, the cells were stained with 50 μg/ml propidium iodide (Sigma) in the dark at 4°C for 10 min. Finally, the samples were monitored by a flow cytometer (NovoCyte, ACEA), and the FACS data were analysed using FlowJo software. All experiments are performed in triplicates.

### 2.11 Apoptosis assay

Apoptosis assay was performed using Annexin V‐FITC/PI apoptosis Kit (KeyGen). Cells were harvested overnight, and then incubated in Annexin V‐FITC and PI for 15 min. The flow cytometry was used to detect cell apoptosis. All experiments are performed in triplicates.

### 2.12 Wound migration assay

Cell migration was assessed via scratch wound assay. The U87 and U251 cells were seeded into 6‐well plates (1 × 10^6^/well) and cultured until confluence. A scratch was made on the cells with a 20‐μl pipette tip. After being rinsed with PBS twice to remove the broken cells, the cells were cultured in DMEM media without FBS. The photographs would be taken at the indicated time to observe and record wound area, respectively. All experiments are performed in triplicates.

### 2.13 Transwell assay

In vitro migration/invasion assay was performed using a 24‐well transwell unit with polycarbonate filters. For cell invasion, the upper chamber of the filter was coated with 500 ng/ml matrigel (Corning), not for cell migration. The lower chamber was filled with DMEM media containing 10% FBS. After incubation at 37°C for 4 h, the matrigel was removed. The U87 and U251 cells were harvested and suspended using serum‐free DMEM media. Then, the cells were plated in the upper part of the transwell (2 × 10^4^ cells/well) and incubated at 37°C for 24 h. The cells were fixed in 4% formaldehyde at room temperature for 20 min, and then stained with crystal violet staining solution for 10–30 min. Following washing three times, the images were captured in three random fields. All experiments are performed in triplicates.

### 2.14 Statistical analysis

The data were expressed as mean ± SEM and analysed using SPSS 19.0 and GraphPad Prism 5.0. Two‐tailed Student's *t*‐test and one‐way anova were used for comparison between two groups and among multiple groups, respectively. *p* < 0.05 was considered statistically significant.

## 3 RESULTS

### 3.1 Identification of read‐through fusion transcript Bcl2l2‐Pabpn1 in rat cells

**FIGURE 1:**
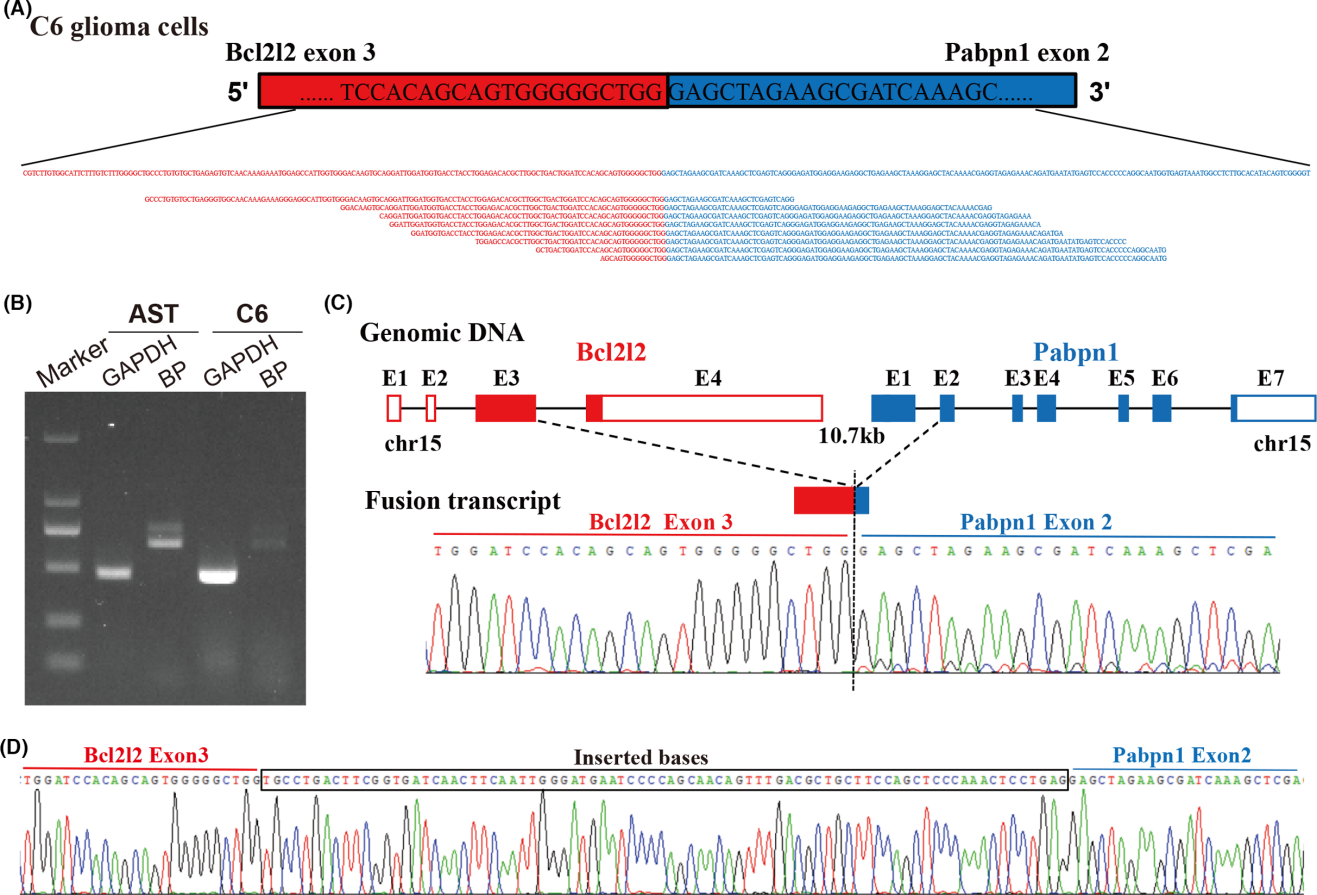
Discovery of Bcl2l2‐Pabpn1 read‐through fusion transcript in rat cells. (A) Bcl2l2‐Pabpn1 fusion transcript was discovered by paired‐end RNA‐seq in C6 glioma cells. RNA‐seq reads are displayed spanning the fusion junction. Bcl2l2 is depicted in red, and Pabpn1 is depicted in blue. (B) A fragment was detected between 500 and 750 bp (663 bp of the theoretical size) both in C6 cells and normal astrocytes (AST) by RT‐PCR and following agarose electrophoresis. 2000 Marker: 2 kb, 1 kb, 750 bp, 500 bp, 250 bp, 100 bp. (C) Sanger sequencing of RT‐PCR products (lower band) indicated an in‐frame fusion of Bcl2l2 exon 3 and Pabpn1 exon 2. The intergenic region of Bcl2l2 and Pabpn1 involved in fusion is about 10.7 kb apart. Upper: gene structures of Bcl2l2 (red) and Pabpn1 (blue). Lower: the junction sequences of the fusion transcript across the fusion point. (D) Sanger sequencing of RT‐PCR products (upper band) suggested the inserted bases between Bcl2l2 exon 3 and Pabpn1 exon 2

Although some studies have reported fusion genes resulting from genomic alterations in glioblastoma,^14^, ^15^, ^16^ read‐through fusion transcripts have not been previously identified in glioblastoma. To discover read‐through fusion transcripts, paired‐end RNA‐seq data from rat C6 glioma cells (C6) and rat primary astrocytes (AST) was analysed using FusionMap.^36^ Interestingly, we found a read‐through fusion transcript: Bcl2l2‐Pabpn1 both in C6 (Figure 1A) and AST cells (Figure S1A). Further RT‐PCR analysis suggested that Bcl2l2‐Pabpn1 chimeric RNA was present in C6 cells and AST cells (Figure 1B). Moreover, Sanger sequencing of the RT‐PCR products (lower band) verified our RNA‐seq data, revealing that exon 3 of Bcl2l2 fused to exon 2 of Pabpn1 in C6 (Figure 1C) and AST cells (Figure S1B). This fusion used canonical splicing site to join the last splice donor of Bcl2l2 to the first splice acceptor of Pabpn1, generating an in‐frame fusion transcript. Considering the intergenic distance between Bcl2l2 and Pabpn1 was small (10.7 kb), it was possible for this fusion transcript that RNA polymerase could start from the Bcl2l2 promoter, and terminate in the Pabpn1 3′UTR region across the intergenic region.^26^ Surprisingly, Sanger sequencing of the RT‐PCR products (upper band) indicated the additional bases which linked Bcl2l2 exon 3 and Pabpn1 exon 2 (Figure 1D). Bcl2l2‐Pabpn1 fusion was expressed widely in human normal and tumour tissues (provided by RefSeq, Dec 2010), suggesting that Bcl2l2‐Pabpn1 read‐through may be a candidate fusion transcript shared by rats and humans.

### 3.2 Verification of read‐through fusion transcript Bcl2l2‐Pabpn1 in human cells

**FIGURE 2:**
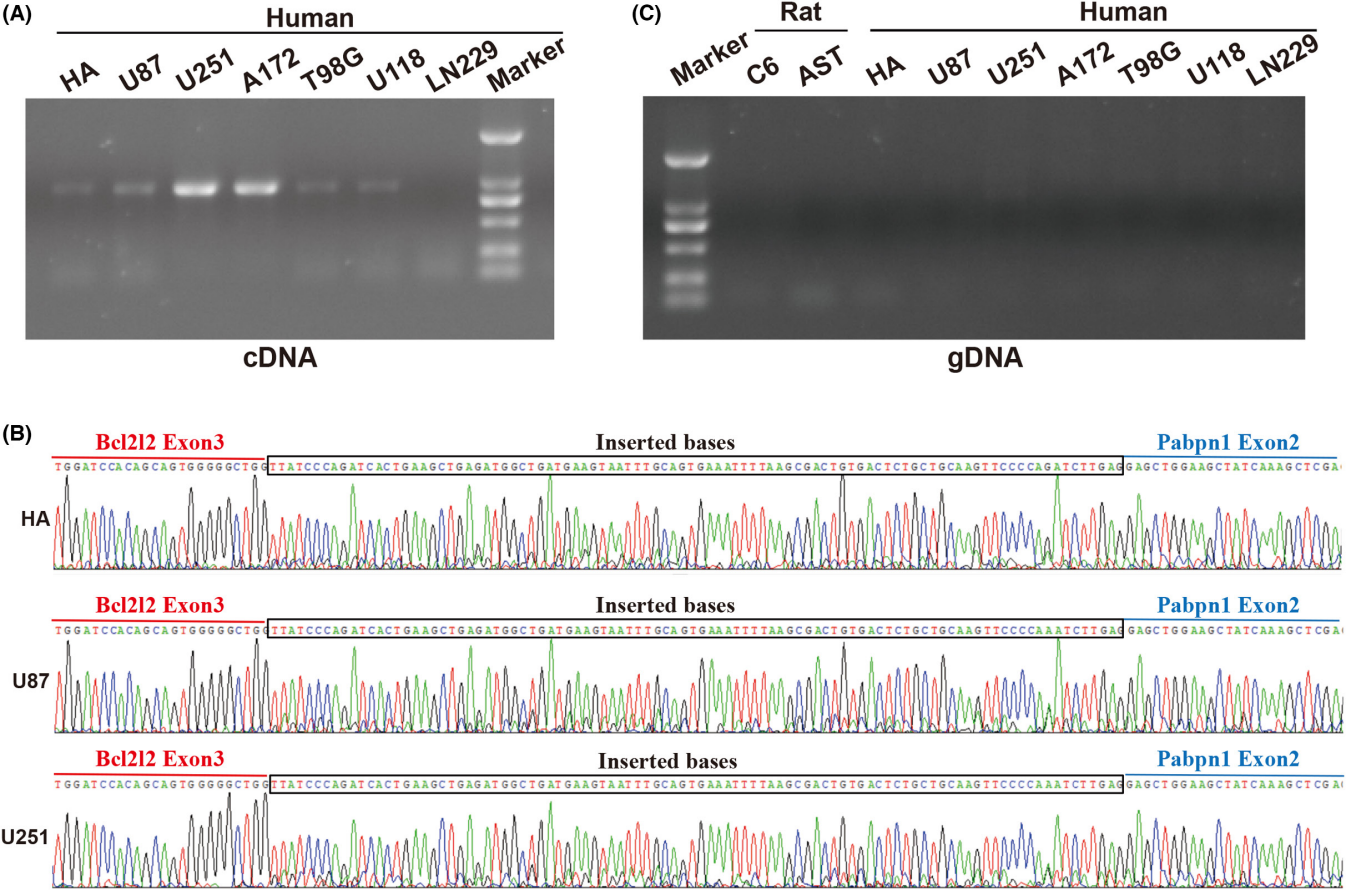
Validation of Bcl2l2‐Pabpn1 fusion in human cells. (A) A clear fragment was detected between 750 bp and 1 kb (821 bp of the theoretical size) in human normal astrocytes (HA) and glioblastoma cell lines except LN229 cells. 2000 Marker. (B) Sanger sequencing of RT‐PCR products confirmed the existence of Bcl2l2‐Pabpn1 read‐through fusion in HA, U87 and U251 cells. Additionally, there are inserted sequences between the exon 3 of Bcl2l2 (red) fused to the exon 2 of Pabpn1 (blue). (C) No corresponding fragment was observed by PCR analysis using genomic DNA (gDNA) as the template in all rat and human samples

Given only a few common fusions between humans and mice,^37^ we evaluated whether this fusion transcript would be expressed in human cells. Excitingly, Bcl2l2‐Pabpn1 fusion was detected both in human glioma cells and human primary astrocytes (HA cells) (Figure 2A). Consistent with the results in rat cells, further Sanger sequencing revealed a fusion between exon 3 of Bcl2l2 and exon 2 of Pabpn1 in human cells (Figure 2B). Interestingly, except for the direct fusing between Bcl2l2 exon 3 and Pabpn1 exon 2, we also observed the indirect linking between them where there were inserted bases in human cells (Figure 2B, Figure S2). Of note, these bases were highly similar in human cells, suggesting that the additional sequences may be common in human cells. These findings were in line with the NCBI database. To determine if genomic rearrangement events are associated with the formation of Bcl2l2‐Pabpn1 fusion, we performed PCR amplification using genomic DNA (gDNA) as a template. Unlike other fusions reported in glioma, Bcl2l2‐Pabpn1 appeared to be generated by RNA processing without DNA rearrangement (Figure 2C). These findings indicated that Bcl2l2‐Pabpn1 could be a common read‐through fusion transcript between glioma and normal cells shared by rats and humans.

### 3.3 Detection of Bcl2l2‐Pabpn1 fusion protein

**FIGURE 3:**
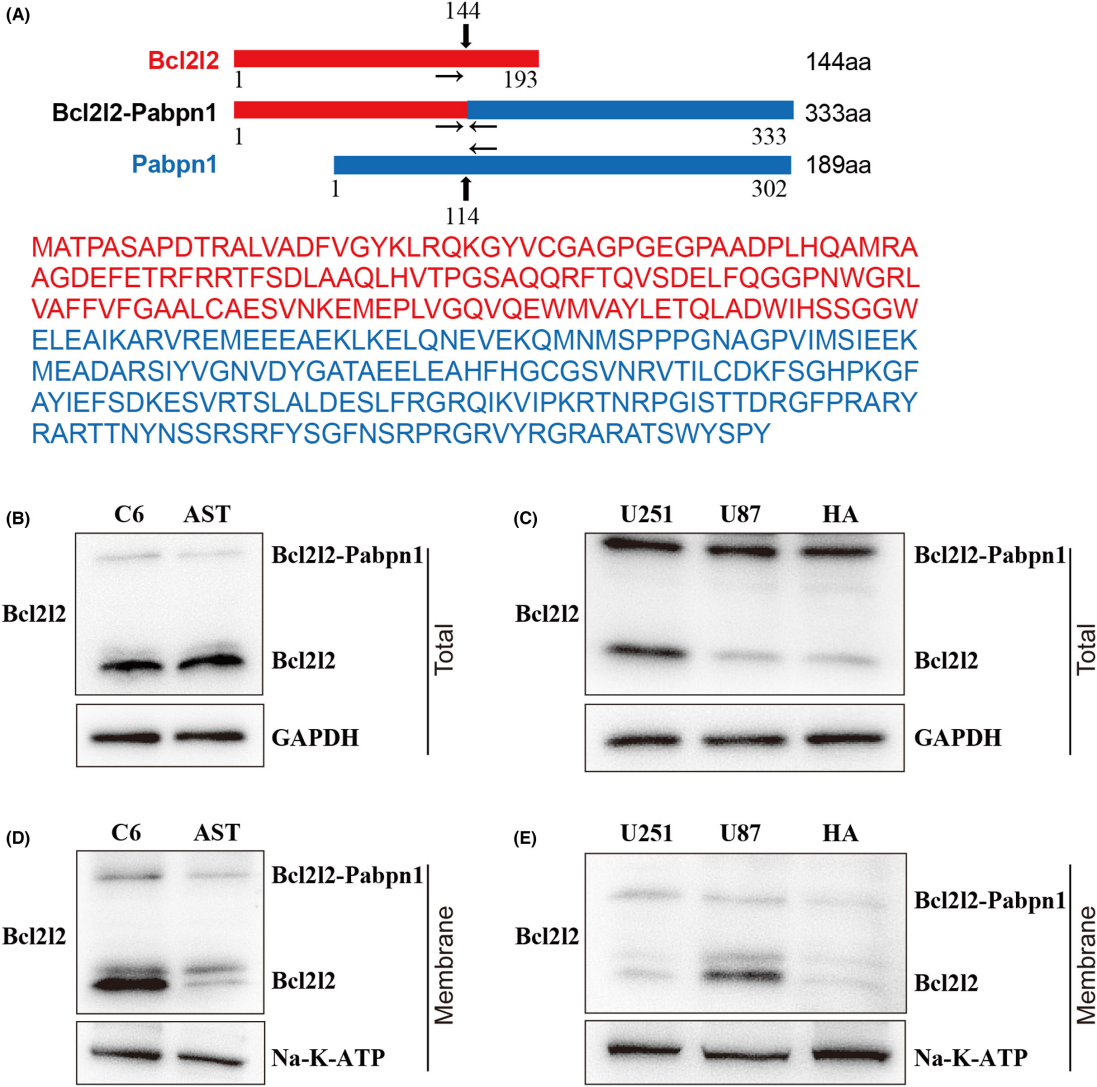
Detection of Bcl2l2‐Pabpn1 fusion protein. (A) Predictive analysis of Bcl2l2‐Pabpn1 fusion protein sequences. Upper: Schematic diagram of the fusion protein. 333 aa of Bcl2l2‐Pabpn1 fusion protein was composed of the first 144 aa of Bcl2l2 (red) and the latter 189 aa of Pabpn1 (blue). Lower: predicted total amino acid sequence of Bcl2l2‐Pabpn1 fusion protein. Western blotting was used to examine the total expression of the fusion protein in rat C6 and AST cells (B), and human HA, U87 and U251 cells (C) with anti‐Bcl2l2 antibody. Western blotting was performed to test the membranal expression of fusion protein in rat C6 and AST cells (D), and human HA, U87 and U251 cells (E) with anti‐Bcl2l2 antibody. In addition to the traditional band of Bcl2l2 (~21 kDa), a larger band is detected besides the predicted size (~37 kDa) in all samples

It has been reported that chimeric RNA transcripts can generate functional fusion proteins.^26^ According to the fusion transcript sequence, we predicted Bcl2l2‐Pabpn1 fusion protein consisted of 333 amino acids, containing 144 amino acids of Bcl2l2 and 189 amino acids of Pabpn1 (Figure 3A). The molecular weight of fusion protein was predicted to be 37 kDa using ExPASy, in accordance with the database (provided by RefSeq, Dec 2010). To examine whether Bcl2l2‐Pabpn1 fusion transcript is translated into the fusion protein, Western blottings were performed using antibodies against one of the partner proteins. Two bands were simultaneously detected at the canonical size of Bcl2l2 and the predicted size of Bcl2l2‐Pabpnl both in rat C6 and AST cells (Figure 3B) with anti‐Bcl2l2 antibody, whereas we observed native band only at expected 49‐kDa of Pabpn1 with anti‐Pabpn1 antibody (Figure S3). Consistently, two targeted Western blottings were tested including the native and fusion proteins using anti‐Bcl2l2 antibody both in human U251, U87 and HA cells (Figure 3C), but only one band was detected at the canonical size (49‐kDa) using anti‐Pabpn1 antibody (Figure S3). Considering Bcl2l2 inserts into the membranes and then acts, the expression of Bcl2l2‐Pabpn1 on the membranes was detected. Expectedly, Bcl2l2‐Pabpn1 was located on the membranes in all cells (Figure 3D,E). These results suggested that Bcl2l2‐Pabpn1 fusion transcript was translated into a fusion protein.

### 3.4 Promotion of glioblastoma progression by Bcl2l2‐Pabpn1 fusion in vitro

**FIGURE 4:**
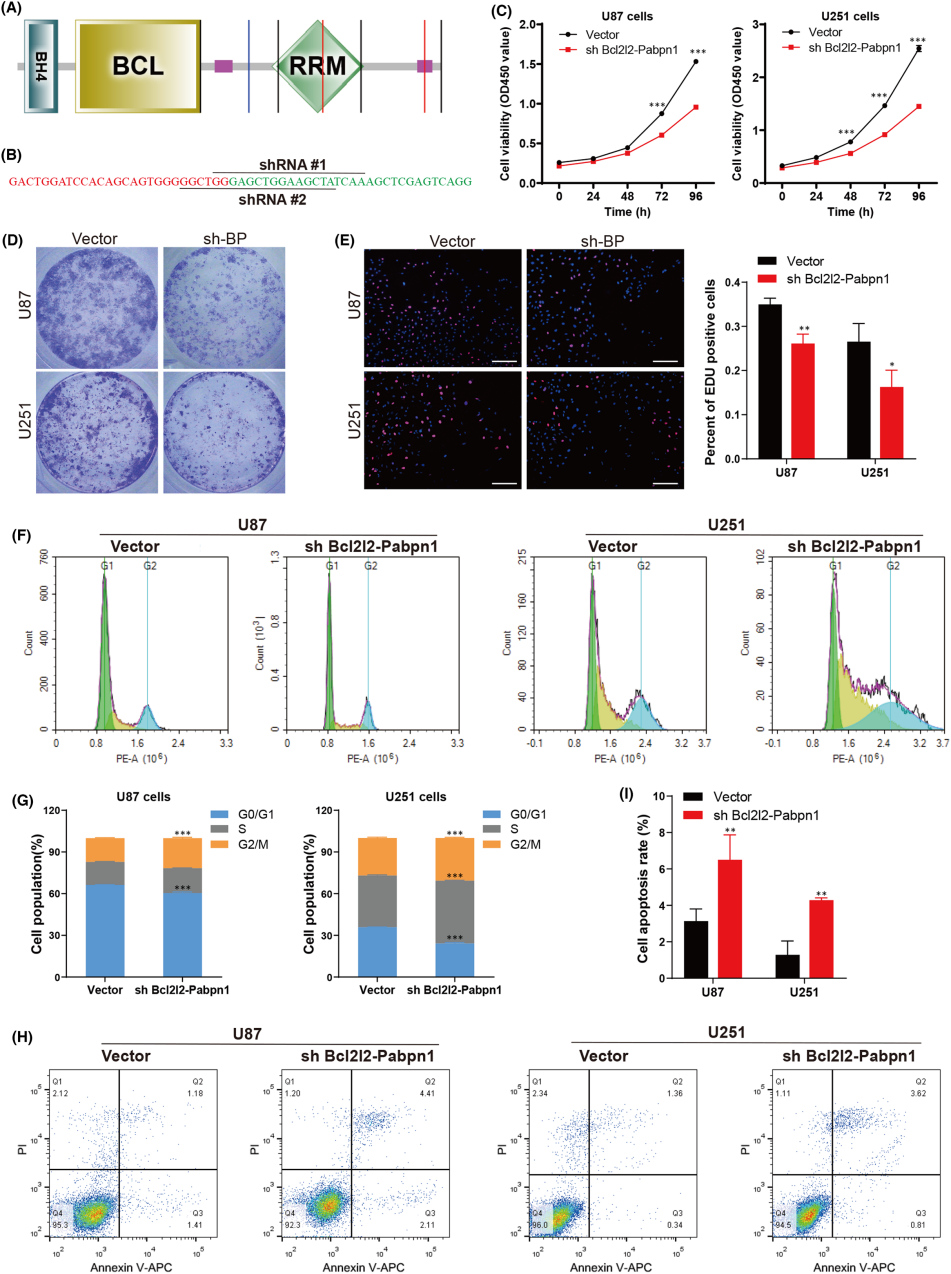
Promotion of cell proliferation by Bcl2l2‐Pabpn1 fusion. (A) Prediction analysis of the domains of Bcl2l2‐Pabpn1 fusion. (B) shRNA sequences targeting Bcl2l2‐Pabpn1 fusion were designed in human cells. (C) CCK8 assay was performed to detect the cell viability. (D) Colony formation assay was conducted to measure the colonizing ability. (E) Cell proliferation was tested by EdU staining. (F, G) Flow cytometry was carried out to assess cell cycle arrest. (H, I) Flow cytometry was used to determine the effect of Bcl2l2‐Pabpn1 on cell apoptosis. *p < 0.05, **p < 0.01, ***p < 0.001

Prediction analysis of the domains indicated that Bcl2l2‐Pabpn1 fusion protein contains three main domains including BH4, BCL domains from Bcl2l2 and RPM domain from Pabpn1 (Figure 4A), suggesting that Bcl2l2‐Pabpn1 maintained the structural characteristics of the Bcl2 family, and may have similar functions to Bcl2l2.

To examine the effects of Bcl2l2‐Pabpn1 on glioblastoma development, we designed two shRNAs (Figure 4B) targeting the fusion junction of Bcl2l2‐Pabpn1 read‐through fusion transcript (NM_001199864.3). U251 and U87 cells, two fusion‐positive glioblastoma cell lines, were transfected with shRNAs. To improve the accuracy of detection, we designed two pairs of primers flanking the fusion junction to examine the efficiency of Bcl2l2‐Pabpn1 knockdown. Only shRNA#1 produced knockdown of Bcl2l2‐Pabpn1 fusion, remaining 40%–50% of the transcript relative to control shRNA (Vector) (Figure S4A). Also, shRNA#1 significantly decreased the protein expression of Bcl2l2‐Pabpn1 (Figure S4B). Then, we stably transfected empty vector and Bcl2l2‐Pabpn1 knockdown lentivirus (sh Bcl2l2‐Pabpn1) in U87 and U251 cells.

**FIGURE 5:**
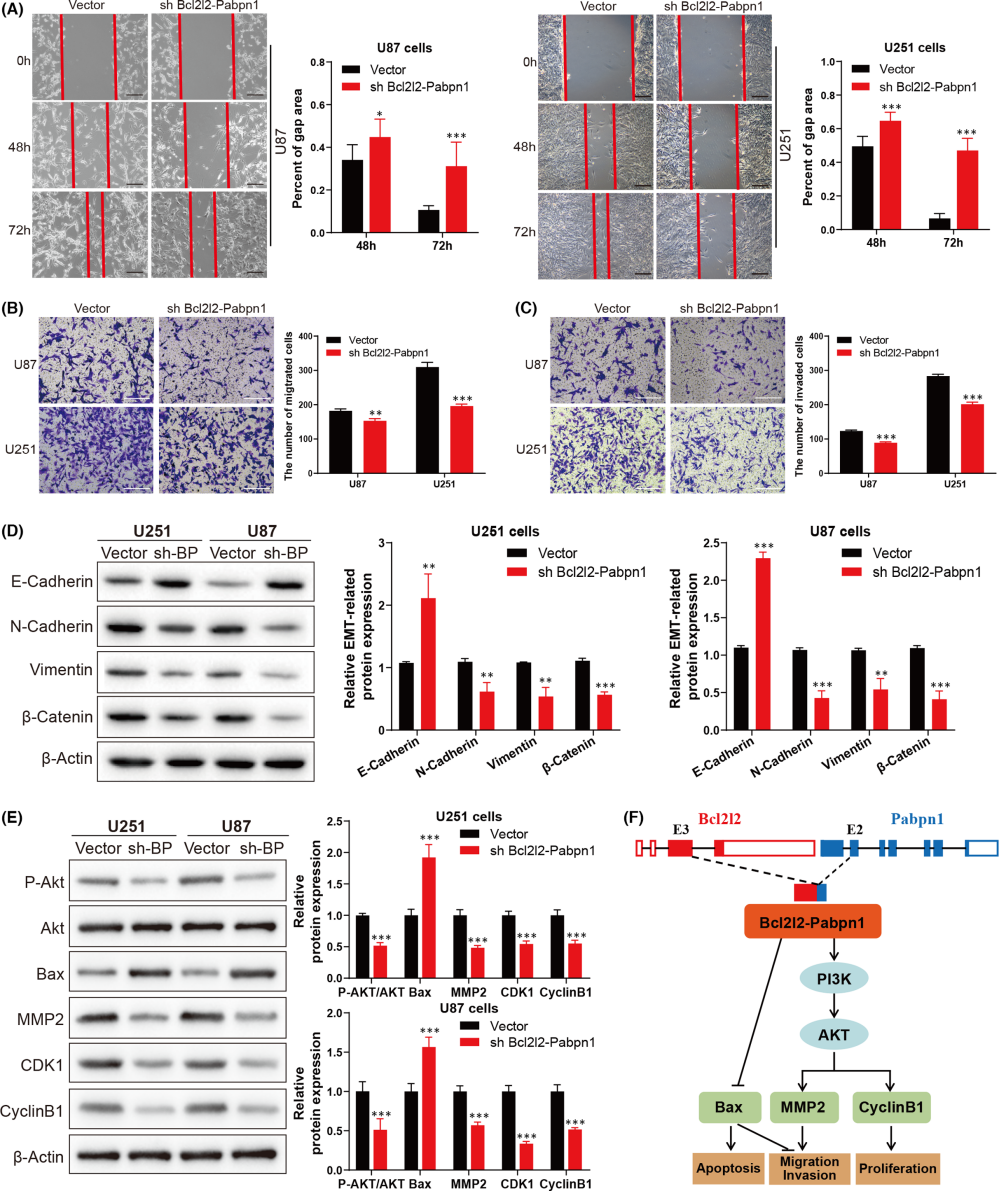
Promotion of cell migration and invasion by Bcl2l2‐Pabpn1 fusion. (A, B) The migration capacity was evaluated by wound‐healing assays and transwell assays without matrigel. (C) The invasion capacity was examined by transwell assays with matrigel. (D) The expression of EMT‐related proteins was analysed when Bcl2l2‐Pabpn1 was knocked down. (E) Western blotting was performed to detect the expression of AKT, Bax, MMP2, CDK1 and cyclinB1. (F) A cartoon summarizing our findings. *p < 0.05, **p < 0.01, ***p < 0.001

To determine the roles of Bcl2l2‐Pabpn1 in glioblastoma progression, functional experiments in vitro were performed. Downregulation of Bcl2l2‐Pabpn1 decreased the cell viability (Figure 4C) and colonizing ability (Figure 4D), compared with the vector group. Furthermore, EDU staining assays showed that cell proliferation was reduced when Bcl2l2‐Pabpn1 was knockdowned in U87 and U251 cells (Figure 4E). Given that the cell cycle plays a vital role in cell proliferation,^38^ flow cytometric analysis was performed to measure the cell cycle distribution. Downregulation of Bcl2l2‐Pabpn1 arrested the cell cycle at the G2‐M phase in glioblastoma cells (Figure 4F,G), indicating that reduced proliferation activity induced by Bcl2l2‐Pabpn1 deficiency might be related to G2‐M phase cell cycle arrest.^39^ Additionally, cell apoptosis was significantly promoted by Bcl2l2‐Pabpn1 knockdown using flow cytometry (Figure 4H,I). We further explored whether Bcl2l2‐Pabpn1 participates in the motility of glioblastoma cells. The results showed that cell migration (Figure 5A,B) was inhibited both in U87 and U251 cells when Bcl2l2‐Pabpn1 was silenced. Furthermore, transwell matrigel assays revealed that Bcl2l2‐Pabpn1 deficiency restricted cell invasion (Figure 5C). Epithelial‐to‐mesenchymal transition (EMT) is closely associated with tumour invasion and metastasis.^40^ Bcl2l2‐Pabpn1 knockdown promoted E‐cadherin expression, and suppressed N‐cadherin, vimentin and β‐catenin expression (Figure 5D). Mechanistically, downregulation of Bcl2l2‐Pabpn1 decreased P‐AKT, MMP2, CDK1 and Cyclin B1 and increased Bax (Figure 5E,F). Collectively, these findings indicated that Bcl2l2‐ Pabpn1 fusion transcript might be a novel regulator of proliferation, migration and invasion in glioblastoma.

## 4 DISCUSSION

Traditionally, gene fusions arising from DNA rearrangement have served as promising biomarkers and therapeutic targets. Some fusion genes have been documented in glioblastoma. Additionally, chimeric fusion transcripts resulting from RNA events are also identified in many cancers.^27^ However, to the best of our knowledge, there are no characterized fusion transcripts in glioblastoma yet.

In the present study, a fusion transcript that fused Bcl2l2 exon 3 to Pabpn1 exon 2 was detected by RNA‐seq. RT‐PCR analysis using the primer flanking fusion points and further Sanger sequencing confirmed the above results. As an important marker of tumours, fusion genes are generally expressed in tumour cells and tissues rather than normal cells and tissues.^26^, ^27^ Unexpectedly, our findings showed Bcl2l2‐Pabpn1 fusion was expressed not only in glioblastoma cell lines but also in normal astrocytes. Although fusion transcripts have been reported as characteristic markers of tumours, they are also present in normal tissues and involved in normal physiology.^19^ A recent research reported that 13 documented tumour fusions were also discovered in normal cells and tissues by RNA‐seq,^37^ indicating that fusion genes were not unique to cancers. Previously, a large number of studies reported that fusion genes are generated by chromosomal rearrangement, with chromosomal translocation being the most common.^5^ However, subsequent studies have discovered that fusion transcripts are produced by cis‐ or trans‐splicing in the absence of genomic rearrangement^41^, ^42^ and are not detectable at the DNA level. Consistently, Bcl2l2‐Pabpn1 fusion was not observed when genomic DNA was used for amplification, implying that Bcl2l2‐Pabpn1 fusion was generated by RNA processing rather than genomic rearrangement.

It is known that human has more chimeric RNAs than mice, and a few fusions are common between humans and mice.^43^ Interestingly, Bcl2l2‐Pabpn1 fusion found in rat cells was also expressed in human glioblastoma and normal cells in our study. The database also shows that this read‐through fusion transcript exists widely in human tumours and normal tissues. These results revealed that Bcl2l2‐Pabpn1 read‐through could be a common fusion transcript in humans and rats. However, differently from the rat cells, there were inserted and highly conserved sequences in human astrocytes and several glioblastoma cell lines. The expression of Bcl2l2‐Pabpn1 needs to be examined in glioblastoma and normal tissues by further experiments.

As a new product of chimerism between two genes, fusion transcript can promote cell proliferation and malignant transformation by forming chimeric oncoproteins.^44^ Indeed, Bcl2l2‐Pabpn1 read‐through led to a fusion protein with BH4, BCL domain of Bcl2l2, and RPM domain of Pabpn1. Therefore, Bcl2l2‐Pabpn1 retained functional domains of Bcl2l2 and could have similar functions. It has been reported that fusion transcript enhances tumour growth and tumorigenesis.^27^, ^45^ In the present study, Bcl2l2‐Pabpn1 knockdown significantly restricted the proliferation, migration, invasion and EMT of human glioblastoma cells. Bax, a major pro‐apoptotic member, mediates cell apoptosis and inhibits cancer development.^46^, ^47^ Bcl2l2 have been documented to promote cell apoptosis, invasion and EMT via antagonizing Bax activity.^30^, ^48^ Previous evidence demonstrates that Bcl2l2 activates the PI3K/AKT signalling and induces MMP2 accumulation to promote cell aggressiveness.^30^, ^32^, ^40^, ^41^, ^42^, ^43^, ^44^, ^45^, ^46^, ^47^, ^48^, ^49^, ^50^ Our work indicated that PI3K/AKT pathway was inactivated, MMP2, CyclinB1 and CDK1 were decreased, and Bax was increased. CyclinB1 and CDK1 are responsible for G2‐M phase transition. However, the molecular mechanisms of tumour‐promoting roles are not fully explored. Additionally, the chimeric fusion RNAs can also affect the cell growth of normal cells,^37^ and whether this read‐through fusion transcript influences normal cell growth needs to be further confirmed.

Collectively, Bcl2l2‐Pabpn1 is a read‐through fusion transcript shared by humans and rats, and present in normal astrocytes and glioblastoma cells. Bcl2l2‐Pabpn1 encodes a fusion protein and promotes glioblastoma progression through blocking Bax and activating PI3K/AKT pathway. Although Bcl2l2‐Pabpn1 fusion is not unique to cancer and is not a candidate biomarker of glioblastoma, our work may provide a new idea for therapeutic intervention of glioblastoma.

## AUTHOR CONTRIBUTIONS

**Lin Zhang:** Conceptualization (lead); data curation (lead); formal analysis (lead); funding acquisition (supporting); investigation (lead); methodology (lead); project administration (lead); software (lead); supervision (lead); validation (lead); visualization (lead); writing – original draft (lead); writing – review and editing (lead). **Dan Wang:** Conceptualization (lead); data curation (lead); formal analysis (equal); funding acquisition (lead); investigation (lead); methodology (equal); project administration (lead); resources (equal); software (lead); supervision (equal); validation (equal); visualization (equal); writing – original draft (equal); writing – review and editing (equal). **Xiao Han:** Conceptualization (equal); data curation (lead); formal analysis (equal); investigation (lead); methodology (equal); software (supporting); validation (lead); visualization (equal); writing – original draft (equal); writing – review and editing (equal). **Xiaoxiao Guo:** Data curation (equal); formal analysis (equal); methodology (equal); validation (lead); visualization (equal); writing – original draft (supporting); writing – review and editing (lead). **Yuanyuan Cao:** Data curation (supporting); formal analysis (equal); investigation (supporting); methodology (supporting); software (equal); supervision (equal); writing – original draft (equal); writing – review and editing (equal). **Ying Xia:** Data curation (supporting); methodology (supporting); software (equal); writing – review and editing (equal). **Dianshuai Gao:** Conceptualization (lead); funding acquisition (lead); project administration (lead); supervision (equal); writing – original draft (lead); writing – review and editing (lead).

## CONFLICT OF INTEREST

The authors declare no potential conflict of interest.

## Supporting information


Appendix S1

## Data Availability

The raw data supporting the conclusions of this article will be made available by the authors, without undue reservation.
