# Supplementary material for: Novel read‐through fusion transcript Bcl2l2‐Pabpn1 in glioblastoma cells
Source: J Cell Mol Med. 2022 Jul 27;26(17):4686–97. doi: 10.1111/jcmm.17481 (PMC9443946; doi:10.1111/jcmm.17481)
Supplement: Supplementary file 1 — Appendix S1 [file JCMM-26-4686-s001.doc]

# Supplementary Tables

***Table S1. Antibody information.***

| **Antibody name** | **Application in this work** | **Catalog number** | **Company** |
| --- | --- | --- | --- |
| Bcl2l2 | Western blotting | DF6292 | affinity |
| Pabpn1 | Western blotting | ab75855 | abcam |
| E-cadherin | Western blotting | 20874-1-AP | Proteintech |
| N-cadherin | Western blotting | 22018-1-AP | Proteintech |
| Vimentin | Western blotting | 10366-1-AP | Proteintech |
| β-atenin | Western blotting | 51067-2-AP | Proteintech |
| Bax | Western blotting | ab32503 | abcam |
| MMP-2 | Western blotting | ab92536 | abcam |
| CDK1 | Western blotting | ab133327 | abcam |
| CyclinB1 | Western blotting | ab32053 | abcam |
| P-AKT | Western blotting | ab38449 | abcam |
| AKT | Western blotting | ab179463 | abcam |

**Supplementary Figures**


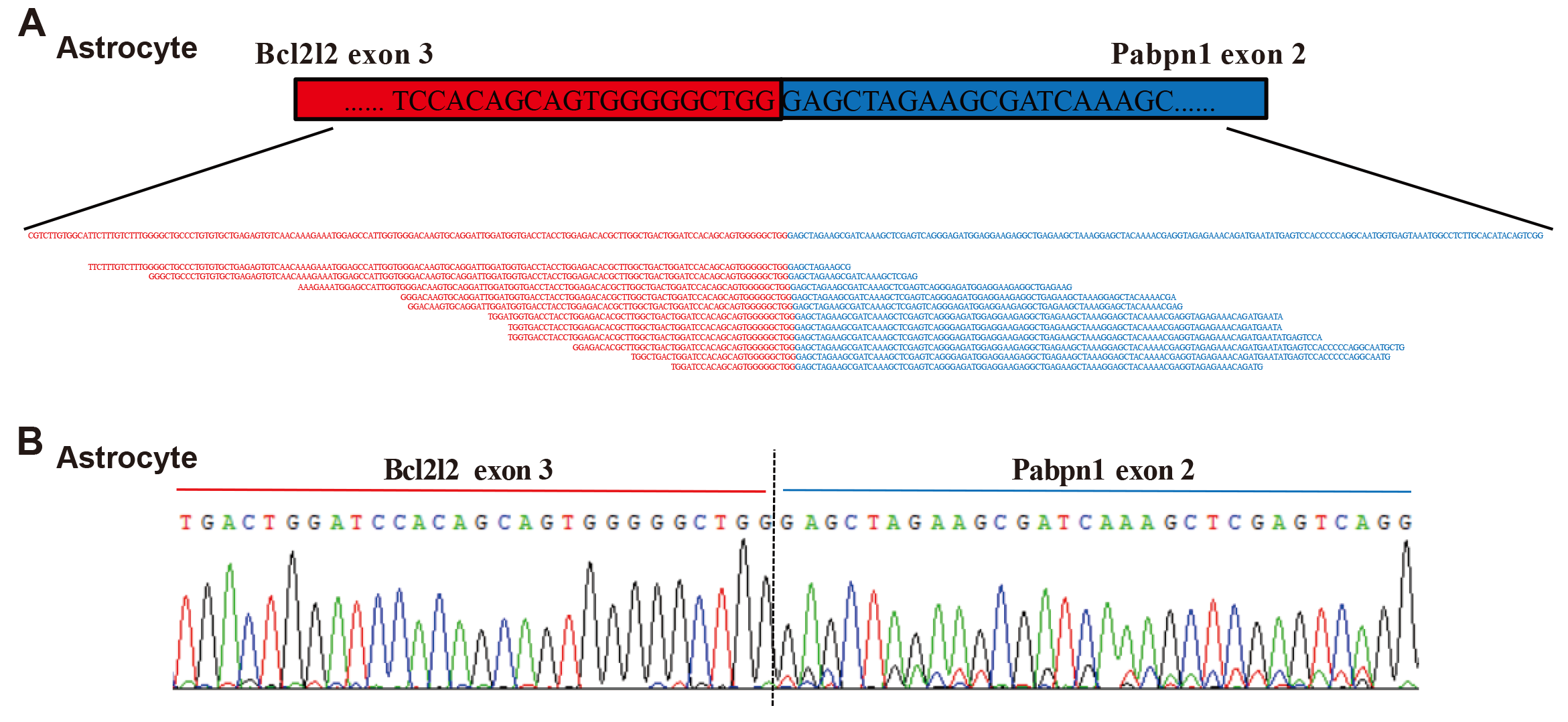


**Figure S1. Detection of Bcl2l2-Pabpn1 fusion in rat astrocytes. (A)** Bcl2l2-Pabpn1 fusion transcript was discovered by RNA-seq in astrocytes. RNA-seq reads are displayed spanning the fusion junction. **(B)** Sanger sequencing of RT-PCR products indicated a in-frame fusion of Bcl2l2 exon 3 and Pabpn1 exon 2. Bcl2l2 is depicted in red, and Pabpn1 is depicted in blue.

**
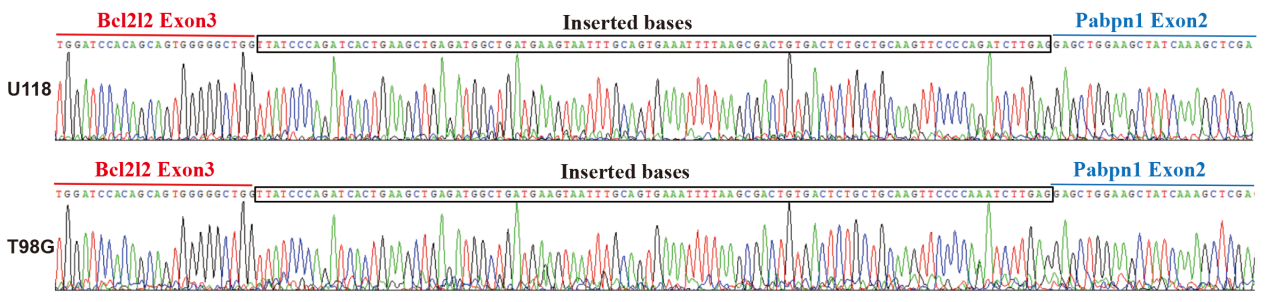
**

**Figure S2. Confirmation of Bcl2l2-Pabpn1 fusion in human glioblastoma cells.** Sanger sequencing of RT-PCR products determined the presence of Bcl2l2-Pabpn1 read-through fusion transcript in U118 and T98G cells. There are inserted sequences between the exon 3 of Bcl2l2 (red) fused to the exon 2 of Pabpn1 (blue) in human cells rather than rat cells.


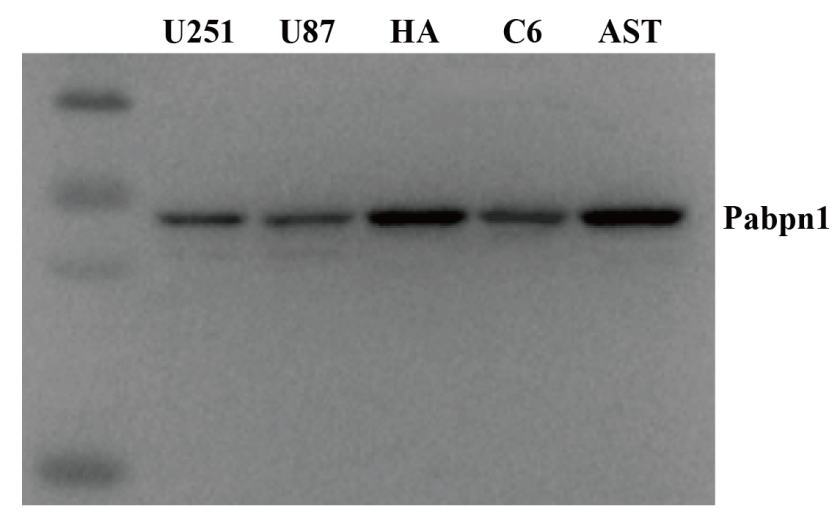


**Figure S3. Detection of Bcl2l2-Pabpn1 fusion protein.** Western blotting was used to test the fusion protein in rat C6 and AST cells, and human HA, U87 and U251 cells with anti-Pabpn1 antibody. Only one native band was observed in ~49kDa.


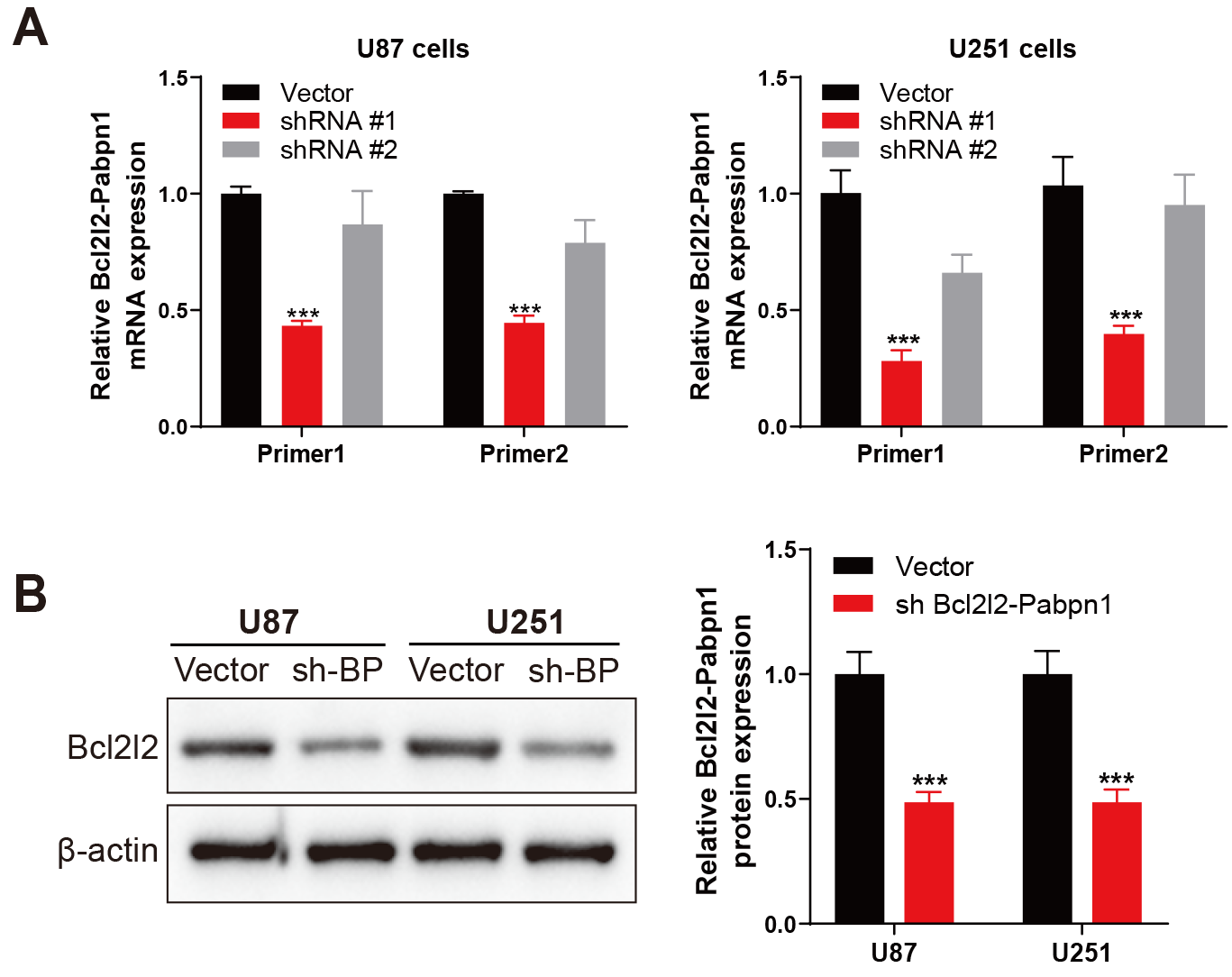


**Figure S4.** **Validation of the efficiency of Bcl2l2-Pabpn1 knockdown. (A)** q-PCR was carried out to measure the mRNA expression of Bcl2l2-Pabpn1. **(B)** Western blotting was used to examine the protein expression of Bcl2l2-Pabpn1. ****p* < 0.001.
